# Is the Motor System Necessary for Processing Action and Abstract Emotion Words? Evidence from Focal Brain Lesions

**DOI:** 10.3389/fpsyg.2015.01661

**Published:** 2015-11-12

**Authors:** Felix R. Dreyer, Dietmar Frey, Sophie Arana, Sarah von Saldern, Thomas Picht, Peter Vajkoczy, Friedemann Pulvermüller

**Affiliations:** ^1^Brain Language Laboratory, Department of Philosophy and Humanities, Freie Universität BerlinBerlin, Germany; ^2^Department of Neurosurgery, Charite University Medicine BerlinBerlin, Germany; ^3^Radboud UniversiteitNijmegen, Netherlands; ^4^Berlin School of Mind and Brain, Humboldt Universität zu BerlinBerlin, Germany

**Keywords:** embodied cognition, category specific impairments, lesion studies, semantic processing

## Abstract

Neuroimaging and neuropsychological experiments suggest that modality-preferential cortices, including motor- and somatosensory areas, contribute to the semantic processing of action related concrete words. Still, a possible role of sensorimotor areas in processing abstract meaning remains under debate. Recent fMRI studies indicate an involvement of the left sensorimotor cortex in the processing of abstract-emotional words (e.g., “love”) which resembles activation patterns seen for action words. But are the activated areas indeed necessary for processing action-related and abstract words? The current study now investigates word processing in two patients suffering from focal brain lesion in the left frontocentral motor system. A speeded Lexical Decision Task on meticulously matched word groups showed that the recognition of nouns from different semantic categories – related to food, animals, tools, and abstract-emotional concepts – was differentially affected. Whereas patient HS with a lesion in dorsolateral central sensorimotor systems next to the hand area showed a category-specific deficit in recognizing tool words, patient CA suffering from lesion centered in the left supplementary motor area was primarily impaired in abstract-emotional word processing. These results point to a causal role of the motor cortex in the semantic processing of both action-related object concepts and abstract-emotional concepts and therefore suggest that the motor areas previously found active in action-related and abstract word processing can serve a meaning-specific necessary role in word recognition. The category-specific nature of the observed dissociations is difficult to reconcile with the idea that sensorimotor systems are somehow peripheral or ‘epiphenomenal’ to meaning and concept processing. Rather, our results are consistent with the claim that cognition is grounded in action and perception and based on distributed action perception circuits reaching into modality-preferential cortex.

## Introduction

A fundamental theoretical debate about the nature of meaning and concepts dominates the cognitive and brain sciences. Classic cognitive psychologists propose that semantic and conceptual processes are carried by a dedicated symbolic semantic system functionally detached from sensory and motor modules and specialized for handling information about meaning and concepts related to signs (e.g., Ellis and Young, 1988). An alternative approach, sometimes referred to by the terms ‘embodiment’ and ‘semantic grounding’, states that meaning is intrinsically related to (or grounded in) action and perception information and processed in the brain by distributed action perception circuits that reach into motor and sensory brain areas (Barsalou, 1999, 2008; Pulvermüller, 2005; Glenberg and Gallese, 2012). Some recent attempts to amalgamate both positions into one integrative proposal either maintain that semantic processing is processed by an amodal system, whereas modality-preferential cortices, such as the sensorimotor areas, play an optional, merely “coloring” role (Mahon and Caramazza, 2008; Caramazza et al., 2014), or they postulate semantic integration in a ‘semantic hub’ (typically placed in temporal cortex) and allow for additional modality-specific semantic centers across the cortex (Patterson et al., 2007; for review, see Binder and Desai, 2011; Kiefer and Pulvermüller, 2012). However, similar to the symbolic systems position, these proposals attribute true semantic processing and related deficits primarily to semantic hub areas. To cite but one relevant statement here: “understanding the word “run” ***occurs in*** modality-independent neural systems” (Bedny and Caramazza, 2011, p. 92; our own emphasis). Therefore, it is not clear whether this type of ‘integrative’ position allows for the explanation of category-specific deficits arising from a focal lesion in one modality-preferential cortical system.

Much recent imaging work has accumulated evidence that the motor cortex (Damasio et al., 1996; Martin et al., 1996; Hauk and Pulvermüller, 2004; Hauk et al., 2004; Pulvermüller et al., 2006) and a range of sensory systems (González et al., 2006; Kiefer et al., 2008; Barrós-Loscertales et al., 2012) become active when words and concepts from different semantic categories are being processed. In particular, the motor system instantaneously activates in a somatotopic fashion when subjects hear or read words semantically related to different parts of the body (Pulvermüller et al., 2005b; Shtyrov et al., 2014), thus arguing against the view that the ‘grounded’ sensorimotor activations may only emerge at a late stage of *post hoc* interpretation and supporting their genuine role in semantic information access. Category-specific semantic activation across the motor system has originally been reported for action-related verbs, but has recently been replicated for nouns semantically related to the mouth and hand (food and tool nouns; Carota et al., 2012). Although some researchers argue some of these effects are difficult to reproduce (Postle et al., 2008; Caramazza et al., 2014), systematic comparison of studies across labs demonstrated good reproducibility (Carota et al., 2012; Kemmerer et al., 2012). Semantically related activation in the motor system has even been reported for abstract words related to emotions (Moseley et al., 2012).

However, although these brain activation studies are consistent with, and confirm predictions of, the grounded-semantic account postulating the relevance of modality preferential areas for semantics, neuroimaging and neurophysiological studies can never proove the functional relevance and necessity of brain areas for cognitive function. To investigate this crucial issue, lesion studies in neurological patients and neurostimulation approaches are necessary.

Here, a range of results has so far been suggestive of a role of sensorimotor systems in semantic processing. For example, Pulvermüller et al. (2005a) applied single TMS pulses to primary hand and foot motor cortex while verbs semantically related to hand or foot actions had to be recognized in a Lexical Decision Task (LDT). As the recognition latencies for hand- and leg-related action verbs was differentially affected by TMS stimulation site (an effect confirmed by a significant interaction of these factors), a causal role of the motor cortex on semantic word type processing was evident. The latter conclusion was also supported by further TMS work in healthy subjects (Willems et al., 2011) and by behavioral experiments in which subjects engaged in motor activity while linguistic-semantic information had to be processed (Glenberg et al., 2008; Witt et al., 2010; Shebani and Pulvermüller, 2013). However, as most of the causal effects of motor activity on semantic processing were manifest in RTs but not accuracies (ACCs), it may still be that the functional role of motor systems for category-specific semantic processing is only relevant for optimizing word processing, but not necessary for it.

Stronger claims about the necessity of modality preferential, including sensorimotor, cortex for semantics can potentially be derived from lesions studies. Important and well-known classic work reported lesion-related category-specific semantic impairments for words related to manipulable objects (Warrington and McCarthy, 1983, 1987), animals and foods (Warrington and Shallice, 1984; for a recent review see also Gainotti, 2010), which were manifest in task ACCs. On closer inspection, the observed patterns of impairments confirm that lesions of regions that include motor areas can lead to selective and pronounced deficits in the processing of action verbs (Damasio and Tranel, 1993; Bak et al., 2001; Neininger and Pulvermüller, 2001, 2003; Arevalo et al., 2012; Kemmerer et al., 2012). Similar results, supportive for theories of embodied cognition, were found after lesions of auditory (Bonner and Grossman, 2012; Trumpp et al., 2013), and visual systems (Gainotti, 2010; Pulvermüller et al., 2010) for the processing of words with auditory or primarily visual semantics. Whereas lesions in modality-preferential sensorimotor cortex brings about deficits in processing action-related words, the granularity of the category-specific deficit is under discussion (see Arevalo et al., 2012; Reilly et al., 2014). At present no evidence exists for a differential involvement of hand- or face-related action words, which can be found amongst verbs (‘write’ vs. ‘chew’) but also amongst the nouns (hand-related tool vs. mouth-related food words) (Arevalo et al., 2012).

Unfortunately, several limitations apply to the majority of previous patient studies. First, the patient populations under investigation typically suffered from large lesions typically caused by stroke or degenerative brain disease. Most of these lesions included motor or sensory cortex but, in addition, other parts of the brain, as in strokes, or even were of diffuse nature, as in motor neuron disease and semantic dementia. Therefore fine grained conclusions about the functional role of specific brain areas in word processing are difficult to derive and it is not entirely clear whether the sensorimotor lesion was indeed the primary cause of the patterns of deficits reported. Second, from a psycholinguistic perspective, the choice of stimulus materials allowed different interpretations of the results. For example, the popular comparison between action-related verbs and object-related nouns frequently led to evidence of a category-deficit, but it is not always clear whether such a deficit is best explained by semantic factors (action- and object-relatedness) or in terms of the lexical (or grammatical) category difference instead (nouns vs. verbs). In addition, relevant psycholinguistic variables such as word length and word frequency were not always matched in previous studies, thus opening further options for alternative explanation of presumed ‘category differences’. However, a small number of recent studies looking at rather focal lesions suggest that auditory and action-recognition systems may also be necessary for processing the semantically related words (Neininger and Pulvermüller, 2001; Campanella et al., 2010; Trumpp et al., 2013).

Although some evidence for a causal and possibly even necessary role of modality preferential cortex for category-specific semantic processing exists, no similar data are available for abstract words whose semantic information is somewhat detached from specific sensory and motor modalities. A major claim held by most symbolic systems accounts, and equally the integrative proposals mentioned above, is that abstract semantic processing is removed from, and does not require, sensory and motor systems of the brain. In contrast, proponents of grounded cognition have argued that, in order to learn the meaning of an abstract word, it is necessary to know at least some concrete semantic instantiations and contexts in which it can be used (Barsalou and Wiemer-Hastings, 2005; Borghi and Cimatti, 2009; Pulvermüller, 2013). At the neuromechanistic level, it has therefore been proposed that abstract meanings, similar to concrete ones, are organized as distributed neuronal circuits including neurons in multimodal and sensorimotor systems, although their links into modality-preferential areas may be weaker than those of concrete conceptual representations. This idea is supported by behavioral (Glenberg et al., 2008) and fMRI findings (Moseley et al., 2012), both indicating an involvement of motor processing in comprehension of abstract words. A strong version of a semantic grounding position thus implies that modality preferential sensorimotor cortex also takes a crucial role in abstract word processing (Glenberg et al., 2008; Havas et al., 2010), but to our knowledge this has so far not been shown with neither neurostimulation, nor lesion approaches. If correct, this position predicts that lesions in modality preferential cortex, and in motor areas specifically, can lead to category-specific semantic deficits in processing abstract words. Positive evidence for this statement would certainly falsify symbolical semantic accounts and most integrative proposals still leaning toward abstract-symbolism, too (Mahon and Caramazza, 2008; Dove, 2009).

On the background of this pre-existing work, the current study addresses the putative necessary role of the modality preferential sensorimotor cortex in the processing of both, action-related and abstract words by examining two patients with focal brain lesions. Although group studies were once claimed necessary for drawing strong conclusions on the brain basis of cognition and language, we would argue that single case reports are indeed suited perfectly well to provide existence proofs for the claimed causality, as they are relevant for the current debate. In addition, some researchers have highlighted the advantages of single case studies, especially if brain localizations of function can strongly differ between individuals – as it is known to be the case for sensorimotor functions (Elbert et al., 1995; Buonomano and Merzenich, 1998) – and the grouping of patients with necessarily non-identical lesions is always debatable (Caramazza, 1986). However, we hasten to add that, whereas case studies can confirm claims about existence (‘there is one case for which it applies that…’), they clearly cannot found general (‘all’) statements.

To overcome the mutual confounding of word semantics and grammatical class, as present in previous patient studies, the current study probed both nouns and verbs separately. This opens the possibility of finding category-semantic deficits that are, in addition, specific to lexical class. With the inclusion of abstract word categories, it becomes possible to investigate whether semantic grounding in modality-preferential cortex applies exclusively to concrete words, or extend to the domain of abstract semantics. To allow conclusions about semantic processing rather than to other stimulus features, semantic categories were matched for a range of psycholinguistic features (see Methods). Word recognition was monitored using a speeded LDT. Performance on this task has previously been shown to be sensitive to aspects of word semantics (Chumbley and Balota, 1984; see also Neininger and Pulvermüller, 2001, 2003). Furthermore, the LDT has important advantages over other tests frequently used in previous studies of semantic category specificity, including, for example, picture naming or categorical classification. These latter tasks require a similar semantic relationship between words and pictures (which, however, differs between concrete and abstract items) and similar perceptual-semantic similarity structure (which differs, for example, between animals and tools), the absence of which limits the scope of their use. In contrast, the LDT offers a straightforward possibility to test performance across word categories differing in their (e.g., abstract vs. concrete) semantics; it has therefore been applied frequently in previous research targeting effects of concreteness and semantic category specificity (e.g., James, 1975; Kroll and Merves, 1986; Jin, 1990; Neininger and Pulvermüller, 2001, 2003; Samson and Pillon, 2004). The rational underlying this research strategy is the following: If semantic processes elicited by one semantic type of words are specifically supported by a given area and if this area is lesioned, the recognition process of the respective word category can be impaired (delayed and/or less accurate). And if a deficit specific for a specific semantic category results from a focal lesion, the lesioned area is a likely key site for processing the affected semantic type. The theoretical background for this prediction is the theory of distributed semantic circuits, according to which neuronal networks with different cortical distributions underlie the processing of different semantic word types (see Pulvermüller, 2013). A focal lesion in an area belonging to the distributional pattern of one semantic word type, but not other word types, would lead to a reduction of the excitatory feedback in the respective category-specific semantic circuits and therefore to delayed and more errorful word recognition. By testing two patients suffering from focal lesions in their frontocentral sensorimotor cortices, this study aims at fine grained conclusions on the functional involvement and necessity of the focal brain areas for the recognition of words from specific semantic categories. Adding abstract words to the stimulus material thereby allows to test whether such a crucial role of these modal areas just applies to the processing of words related to concrete concepts or even extends to abstract words.

## Materials and Methods

### Patients and Clinical Examination

#### Patient HS

**FIGURE 1:**
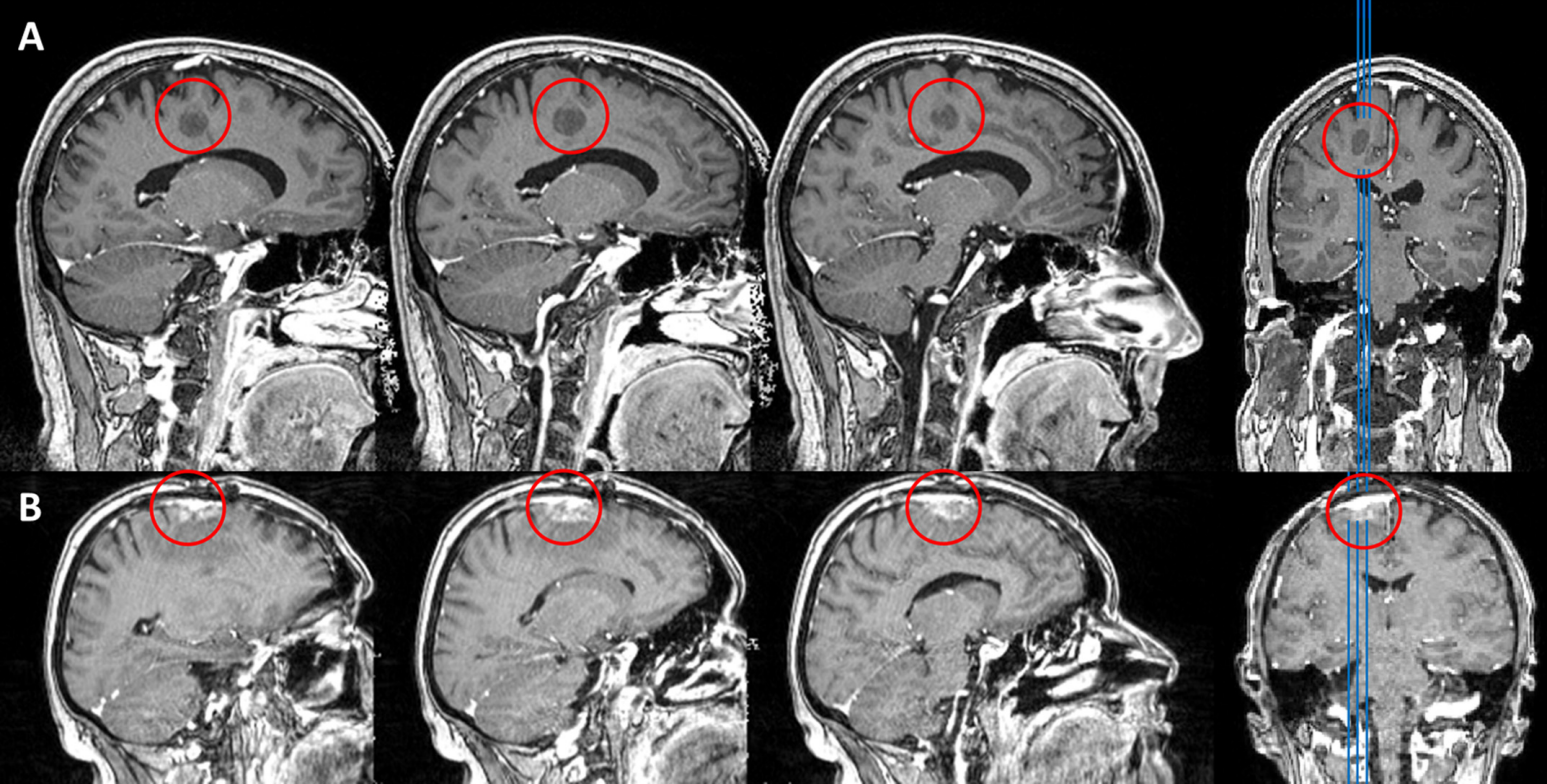
**T1 weighted MRI MPRAGE sequences of patients HS **(A)** and CA **(B)**.** Lesion sites are marked with a red circle.

Patient HS was a 41 years old man, with a singular focal precentral lesion, situated directly inferior to the left hand motor cortex. HS was a native, monolingual German speaker and right handed (LQ = 80), with a total of 18 years of formal education and was serving in the military at the time of testing. Following biopsy, HS’ lesion was diagnosed to be the single residual core of an Acute Disseminated Encephalomyelitis (ADEM) of 18 mm in diameter. Fiber tracking on Diffusion-Tensor-Imaging (DTI) data, using hotspots of an nTMS guided motor mapping procedure as seed regions (see Frey et al., 2012 for details) revealed his lesion to be situated in the precentral gyrus, half a centimeter away from the pyramidal tract of the hand motor cortex. A T1 weighted MRI scan of this lesion is shown in **Figure 1A.** At the time of language testing, neurological examination revealed mild paresis of the right arm and leg (grade 4, i.e., movement against external resistance, but less than normal), but no other cognitive or language impairment.

#### Patient CA

Patient CA was a 52 years old woman with a single lung cancer metastasis (histology: adenocarcinoma as non-small-cell lung carcinoma) in the superior frontal gyrus, affecting the supplementary motor area (SMA) of the left hemisphere, as shown in **Figure 1B.** The patient had been under chemotherapy for three cycles, underwent radiation therapy for two cycles and a first extirpation of the tumor had been performed 6 month prior to testing. All therapeutic measures did not result in control of the solitary cerebral metastasis. Due to growth of the tumor (with an extent of roughly 1.2 cm × 0.9 cm × 2 cm), indication for additional surgical removal was yielded at the time of testing. History revealed hypertension, chronic obstructive pulmonary disease and the regular administration of Pregabalin and Amitriptylin. CA was right handed (LQ = 80 at the time of testing) and a native, monolingual German speaker with 12 years of formal education and had been working as a chef pre-morbidly. CA did not report any sensory, motor, cognitive or language deficits and neurological examination did not reveal any impairments on those dimensions at the time of testing.

#### Control Participants

A group of 21 participants (five males) without neurological records served as control sample for the LDT paradigm. On average, controls were 40.7 years (*SD* = 18.7 years) old at the time of testing, with an age range from 18 to 79 years, covering that of the two neurological patients. Likewise, years of formal education were similar to CA and HS, spanning between 11 and 24 years, with an average of 16.5 years (*SD* = 3.5 years).

Both, patients and healthy control participants, provided written informed consent prior to participating in the study and procedures were approved by the ethics committee of the Charite University Hospital, Berlin, Germany.

### Paradigm

As critical test, a speeded LDT was carried out, as explained below. To assess clinical language proficiency the Token Test, and the repetition, naming and language comprehension subtests of the “Aachener Aphasie Test”, or AAT, a standardized German aphasia test battery (Huber et al., 1983), were applied. Handedness was tested using the Edinburgh Inventory (Oldfield, 1971).

### LDT Stimuli

Hundred sixty nouns and 160 verbs were presented, along with 320 matched pseudo-words. Each of the lexical/grammatical categories included 40 stimuli from 4 semantic groups or categories. Among the nouns, there were words used to speak about tools, food items, animals, and abstract-emotional entities. The semantic category groups of the verbs included words typically used to speak about actions performed with parts of the face (e.g., “kauen”, *to chew*), hand (e.g., “greifen”, *to grap*), or leg (e.g., “rennen”, *to run*) and about abstract concepts (“hassen”, to *hate;* see Supplementary Data for a complete overview of word stimuli).

Within each lexical category or grammatical word class, all semantic category groups were matched for a range of lexical and sub-lexical psycholinguistic variables, as determined by the dlex corpus (Heister et al., 2011). Matching was achieved for word length, number of syllables, phonological stress, normalized lemma frequency, character bigram frequency, character trigram frequency, initial character-, initial character bigram-, and initial character trigram frequency as well as for number of orthographic neighbors in terms of Coltheart’s and Levenshtein’s N. *F/t*-tests did not reveal differences between semantic category groups for any of these psycholinguistic variables (all *p* >0.05, see **Tables 1** and **2** for details).

**Table 1:** Matching on psycholinguistic variables between semantic classes in nouns.

	Nouns						
	
	Abstract-emotional		Animals		Foods		Tools		
					
Variables	*M*	*SD*	*M*	*SD*	*M*	*SD*	*M*	*SD*	*p*
Lemma Frequency p. Mio.	8.41	5.56	7.26	5.47	5.95	7.74	6.86	5.97	0.37
Length	5.7	1.4	5.5	1.66	5.78	1.37	5.93	1.47	0.64
Number of Syllables	1.7	0.46	1.7	0.46	1.78	0.42	1.88	0.33	0.21
Character bigram frequency p.Mio.	216786	101041	243304	123503	210825	120275	250937	145228	0.39
Character trigram frequency p.Mio.	120397	67480	148481	68331	124296	78409	125515	88827	0.35
Initial character frequency p.Mio.	12171	5742	13974	5816	14427	6163	14992	7248	0.21
Initial bigram frequency p.Mio.	2346	1926	2349	1901	1956	2000	2599	2321	0.57
Initial trigram frequency p.Mio.	414	926	748	1703	473	1262	913	1882	0.4
Coltheart neighbors frequency p.Mio.	126	488	82	269	28	74	56	117	0.47
Coltheart’s N	6.08	6.07	7	6.65	6.01	5.77	7.16	5.7	0.76
Levenshtein neighbors frequency p.Mio.	256.35	1272.33	165.29	547.99	147.26	594.08	61.24	118.91	0.72
Levenshtein N	8.63	7.47	9.9	8.26	8.79	7.32	10.36	6.72	0.67


*P-values denote results from one-way ANOVAs on the effect of semantic category.*

**Table 2:** Matching on psycholinguistic variables between semantic classes in verbs.

	Verbs						
	
	Abstract		Face		Leg		Arm		
					
Variables	*M*	*SD*	*M*	*SD*	*M*	*SD*	*M*	*SD*	*p*
Lemma frequency p. Mio.	25.95	26.71	28.32	57.01	28.97	67.23	26.26	35.18	0.99
Length	6.68	1.29	6.98	1.33	7.08	1.35	6.83	1.24	0.54
Number of syllables	2	0	2	0	2	0	2	0	1
Character bigram frequency p.Mio.	544785	123466	536853	104691	534851	151015	530002	123341	0.96
Character trigram frequency p.Mio.	367231	73446	366884	58928	359082	65621	369878	55976	0.89
Initial character frequency p.Mio.	30979	27868	25033	18439	28458	18747	28579	25280	0.71
Initial bigram frequency p.Mio.	4375	8943	3245	3407	3098	3690	3149	2864	0.67
Initial trigram frequency p.Mio.	1368	2173	1155	2200	1012	1880	1320	2280	0.87
Coltheart neighbors frequency p.Mio.	145	227	61	135	104	347	87	262	0.51
Coltheart’s N	5.36	4.08	5.25	4.32	4.38	3.40	4.94	3.5	0.66
Levenshtein neighbors frequency p.Mio.	164.41	233.91	103.64	189.97	114.06	363.51	92.55	262.01	0.65
Levenshtein N	8.47	5.92	7.73	5.14	6.51	4.64	7.33	4.18	0.37


*P-values denote results from one-way ANOVAs on the effect of semantic category.*

In addition, an equal number of pronounceable pseudo-words was generated on the basis of the proper words using the ‘Wuggy’ software (Keuleers and Brysbaert, 2010). These pseudo-words were chosen to be not homophonous to proper words and to match all proper word categories, both combined and individually, in their sub-lexical psycholinguistic properties of average word length, number of syllables, character bigram frequency, character trigram frequency, initial character frequency, and initial bigram frequency (all *p* > 0.05, see **Table 3** for details). To further mimic appearance of proper words, pseudo-nouns all started with a capital letter and pseudo-verbs all ended in the “-en” suffix, consistent with German noun and verb orthography and morphology.

**Table 3:** Matching on psycholinguistic variables between real and pseudo-words.

	Real words		Pseudo-words		
			
	*M*	*SD*	*M*	*SD*	*p*
Character bigram p.Mio. (Sum)	383542	197428	378278	189726	0.73
Character trigram p.Mio. (Sum)	247720	137424	245700	135682	0.85
Initial character p.Mio	21076	18206	22311	21276	0.43
Initial bigram p.Mio	2890	4051	2476	3274	0.16
Mean bigram p.Mio	51299	22628	51973	23000	0.71
Mean trigram p.Mio.	29493	15449	29945	15739	0.71
Length	6.31	1.5	6.12	1.3	0.08


*P values denote results of *t*-test between both stimulus types.*

**FIGURE 2:**
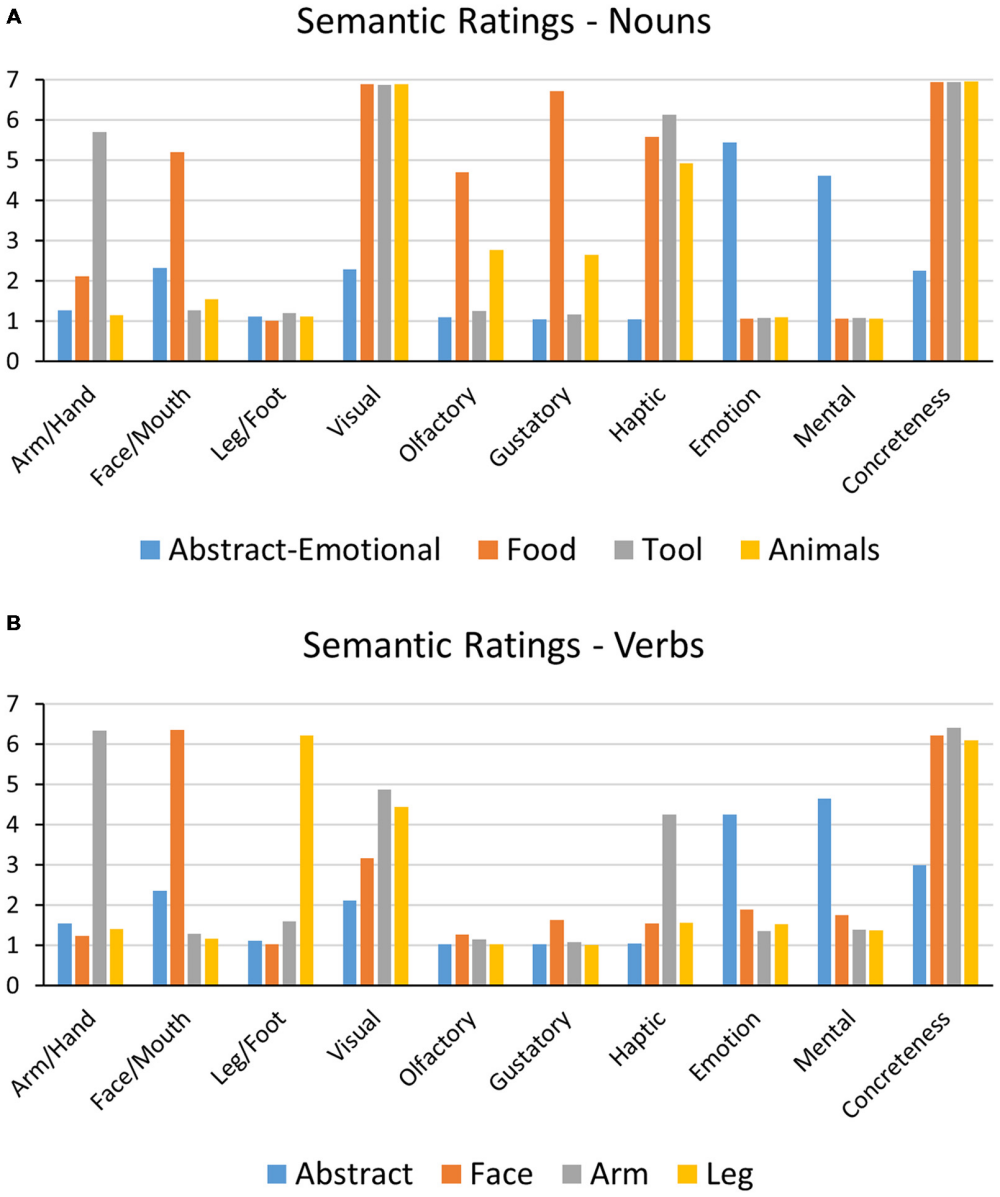
**Average semantic ratings for noun **(A)** and verb **(B)** categories, given on a scale from 1 (no semantic relation) to 7 (very strong semantic relation)**.

To empirically evaluate the semantic properties of the word stimuli, semantic ratings were collected from 20 healthy participants (monolingual native speakers of German aged 18–28) before the main experiment. Similar to previous studies (Pulvermüller et al., 2001; Hauk and Pulvermüller, 2004), semantic ratings were expressed on a Likert scales ranging from 1 (no relation) to 7 (strong relation). Each word was rated for its semantic relatedness to hand/arm-, face/mouth-, leg/foot actions, to visual, olfactory, gustatory, and haptic/tactile perceptions, as well as to emotions and mental processes. Ratings of concreteness and word familiarity were also obtained. The concreteness scale was thereby designed with the poles of high abstractness (1) to high concreteness (7). For inclusion into an effector-specific action word category (action verbs and tool/food nouns), words had to achieve an average rating above the neutral mid-point of four for the related question while being rated lower on all other action semantic scales. For animal nouns and abstract words, all action ratings were <4, with abstract items also rating <4 on concreteness and perceptual scales, but >4 on the scale for relation to mental processes. In addition, all abstract-emotional nouns, and also the majority of abstract verbs had strong emotional connotations with values >4 on the respective semantic scale. Semantic ratings for all categories are shown in **Figure 2**.

### LDT Procedures

Participants were seated approximately 70 cm in front of a computer screen and were instructed to decide whether or not a word flashing on screen resembles a meaningful German word, or a pseudo-word instead. Responses were given via left hand mouse clicks, to assure that responses were not affected by possible motor impairments caused by left hemispheric lesions. Each trial started with a presentation of a central fixation cross. Its presentation time was pseudo-randomly varied between 2250 and 2750 ms (2500 ms on average) and it was followed by an acoustic beep signal of 200 ms length. 800 ms after the offset of this acoustic signal, the fixation cross disappeared and a word was presented tachistoscopically in the center of the screen for 130 ms. After word offset, the screen remained blank until a response was given, or for a maximum of 3000 ms after which the central fixation cross re-appeared. All stimuli were printed in black letters on a light gray background, using monospaced Courier New font with a font size of 13.5 and were spanning a maximum of 2° horizontal and 0.6° vertical visual degree.

Each test session started with 10 practice trials for the LDT, which applied stimuli that were not used in the actual experiment. Those trials were repeated until a task ACC of 80% was achieved, to assure that participants were sufficiently familiarized with task procedures.

The LD experiment was split up into 8 blocks, each including 80 letter strings, five words from each of the eight lexico-semantic categories as well as 20 pseudo-nouns and 20 pseudo-verbs. In addition, two words were presented as filler items at the beginning of each block, which were excluded from analysis. Each block lasted between 6 and 8 min, depending on participants’ response speed. Between experimental blocks, participants were offered breaks.

Following the LDT testing, patients conducted the AAT subtests in the following order: Token Test, Verbal Repetition, Naming and Comprehension. To save time, subjects who performed <7 corrected error points on the Token Test (no aphasia diagnosis) were only given the most difficult part of the other subtests and if their performance was flawless, the rest of the subtest was omitted. On average, the whole aphasia test battery could be conducted within 20 min. Each test session was thereafter concluded by the Edinburgh Handedness Inventory and the basic demographics questionnaire.

### Data Analysis

#### Healthy Control Participants

Lexical Decision Task analyses were conducted separately for noun and verb categories. Note again that all noun categories were matched with each other with regard to psycholinguistic variables, and the same applied for verb categories, but it was not possible to match across lexical (grammatical) categories. To allow response bias corrected comparisons with patients, task ACCs for individual lexico-semantic categories were converted into *d*′ scores. To calculate *d*′ values for each lexico-semantic group of nouns (verbs), each category’s hit rate and the overall false positive rate of the entire lexical (i.e., either pseudo-noun or pseudo-verb) category was used (see also Pulvermüller et al., 2010). Resultant *d*′scores were compared between semantic categories using by-subject repeated measures analyses of variance (ANOVAs), by-item ANOVAS, and *t*-tests with Bonferroni correction for *post hoc* comparisons. Further testing was done to compare the entire noun and verb groups against each other.

Reaction Times for correct responses were corrected for individual outliers >2 standard deviations away from the mean. After correction, average RTs for each lexico-semantic category and individual participant were calculated and performance between semantic categories was compared separately for nouns and verbs. By-subject and by-item repeated measures ANOVAs were then used for overall analyses and *t*-tests for planned comparison testing. An additional analysis step compared the performance between nouns and verbs with repeated measures ANOVAs on *d′* and RT results.

#### Patients

Raw AAT scores were calculated, converted into normalized scores and compared to control samples according to the tests’ instructions.

Lexical Decision Task ACCs for individual lexico-semantic categories were converted into *d′* scores as described above, for each patient individually. We tested for general performance differences between semantic groups within each lexical/grammatical category. To this end, ACC (here expressed as number of hits and misses) was compared using χ^2^- and, in case of insufficient cell sizes (*n* < 5) Fischer’s Exact Tests. In case those tests indicated significant differences, χ^2^ tests with Bonferroni correction were conducted once for each semantic category versus the combined other categories within one grammatical word class (four comparisons). In case of significantly different semantic noun categories, a second set of analyses compared each action or abstract category against the reference category of non-action animal nouns (three comparisons). For completeness, all categories were finally pairwise compared against each other (six comparisons). Note again that analyses were done separately for nouns and verbs.

In the analysis of RT of correct responses, individual outliers >2 standard deviations away from the mean were first removed and the corrected single trial RTs were analyzed for effects of semantic word category using by-item ANOVAs and *t*-tests with Bonferroni correction.

To test whether differences across semantic categories in a specific patient can indeed be considered to be abnormal compared with performance differences between categories seen in the control sample revised standardized difference tests (RSDT; Crawford and Garthwaite, 2005) were conducted as *post hoc* tests. The RSDT resembles a derivate of the *t-*test, specifically designed to relate performance differences of individual patients directly to results of a group of control participants. To account for the inflated Type II error rate of the RSDT, these additional *post hoc* tests were one-tailed (see Crawford and Garthwaite, 2006 for discussion).

Furthermore, to test for effects of grammatical class, ACC performance was compared between all nouns and verbs using the χ^2^ test and with ANOVAs on corrected RTs, respectively, in a separate analysis.

## Results

### AAT

No patient exhibited aphasic language impairments, as the AAT scores fell well within the range of healthy control population performance. Individual results for each patient and subtest are listed in **Table 4.**

**Table 4:** Performance of patients in AAT subtests given in *T*-Scores.

	*T*-Scores	
	
	CA	HS
Token test^∗^	73	73
Verbal repetition^∗^	74	74
Picture naming	80	80
Language comprehension	78	73
Average	76	75


*Tests marked with an ^∗^were conducted in an abbreviated version.*

### LDT

#### Healthy Control Subjects

A repeated measures ANOVA on *d′* scores did not reveal any significant differences between semantic noun categories [*F*(3,60) = 1.59, *p* >0.1, η^2^ = 0.08, n.s.] or across verbs subtypes [*F*(3,60) = 1.56, *p* <0.1, η^2^ = 0.08, n.s.]. However, RTs differed significantly across both semantic categories of nouns [*F*(3,60) = 21.4, *p* <0.001, η^2^ = 0.52] and verbs [*F*(3,60) = 8.3, *p* <0.001, η^2^ = 0.29]. This pattern of results was confirmed with additional item-wise ANOVAs on *d*′ [Nouns: *F*(3,159) = 0.32, *p* = 0.81, η^2^ = 0.01; Verbs: *F*(3,159) = 0.76, *p* = 0.52, η^2^ = 0.01] and RT [Nouns: *F*(3,159) = 7.95, *p* < 0.001, η^2^ = 0.13; Verbs: *F*(3,159) = 3.76, *p* = 0.01, η^2^ = 0.07]. For nouns, Bonferroni corrected *post hoc t*-tests revealed that RTs for abstract-emotional (*M* = 693 ms, *SE* = 15 ms) and tool words (*M* = 689 ms, *SE* = 15 ms) were significantly longer than for food (*M* = 653 ms, *SE* = 14 ms) and animal words (*M* = 662 ms, *SE* = 16 ms, all *t*(20) > = 5, *p* < 0.001, Cohen’s *d* > 1). *Post hoc* tests conducted on verbs showed RTs for hand verbs (*M* = 683 ms, *SE* = 19 ms) to be significantly shorter than for abstract [*M* = 710 ms, *SE* = 17 ms, *t*(20) = 3.5, *p* = 0.01, Cohen’s *d* = 0.77] and face- [*M* = 704 ms, *SE* = 18 ms, *t*(20) = 3.3, *p* = 0.02, Cohen’s *d* = 0.72] as well as leg-related action verbs [*M* = 720 ms, *SE* = 18 ms, *t*(20) = 3.9, *p* < 0.01, Cohen’s *d* = 0.86]. Overall, ACCs in terms of *d′* values for nouns and verbs were both high, with a significant advantage of nouns (*M* = 3.9, *SE* = 0.09) over verbs [*M* = 3.6 *SE* = 0.13, *t*(20) = 3, *p* < 0.01, Cohen’s *d* = 0.65]. RTs results showed a similar pattern, with RTs for nouns (*M* = 674 ms, *SE* = 14 ms) being significantly shorter than for verbs [*M* = 704 ms, *SE* = 18 ms, *t*(20) = 6.4, *p* <0.001, Cohen’s *d* = 1.17].

#### Patient HS

Analysis of ACC revealed a significant difference in task performance between the noun categories (χ^2^ = 10.45, *df* = 3, *p* = 0.01, Cramer’s *V* = 0.26) with performance on tool nouns (ACC = 0.83) being more impaired than that the other three categories combined (ACC = 0.97 on average, χ^2^ = 9.4, *df* = 1, *p* = 0.02, Cramer’s *V* = 0.24). When comparing tool nouns against the reference category of non-action related animal nouns, a significant difference emerged [χ^2^ = 7.67, *df* = 1, *p* = 0.036, Cramer’s *V* = 0.31] For verbs no significant differences in ACC between semantic categories was observed (χ^2^ = 2.11, *df* = 3 *p* = 0.64, Cramer’s *V* = 0.14). The ANOVA on RTs did not show significant differences between categories for either nouns [*F*(3,136) = 0.62, *p* > 0.1, η*^2^* = 0.01, n.s.] or verbs [*F*(3,137) = 0.62, *p* > 0.1, η*^2^* = 0.01, n.s.]. As the RT distribution for verbs hinted a positive skew (Skewness = 0.84, *SE* = 0.2), the corresponding ANOVA was repeated on log-transformed data, but again did not hint significant differences between verbs categories [*F*(3,137) = 0.59, *p* = 0.62, η^2^ = 0.01]. This pattern of results was replicated when comparing HS’ performance with that of healthy controls. HS performance fell within the healthy range (mean ± 2 SDs) in terms of ACC and his RTs on verbs even tended to be faster than those of healthy control subjects. In contrast, for tool words, HS’s ACCs were more than 2 SDs away from the mean of the control group, thus indicating significant slowing and therefore further confirming a selective impairment for tool nouns. *Post hoc* RSDT results confirmed this observation, as the difference in ACCs between animal and tool nouns was significantly more severe in patient HS than in the control sample [*t*(20) = -2.72, *p* = 0.008, Z-DCC = -2.77].

**FIGURE 3:**
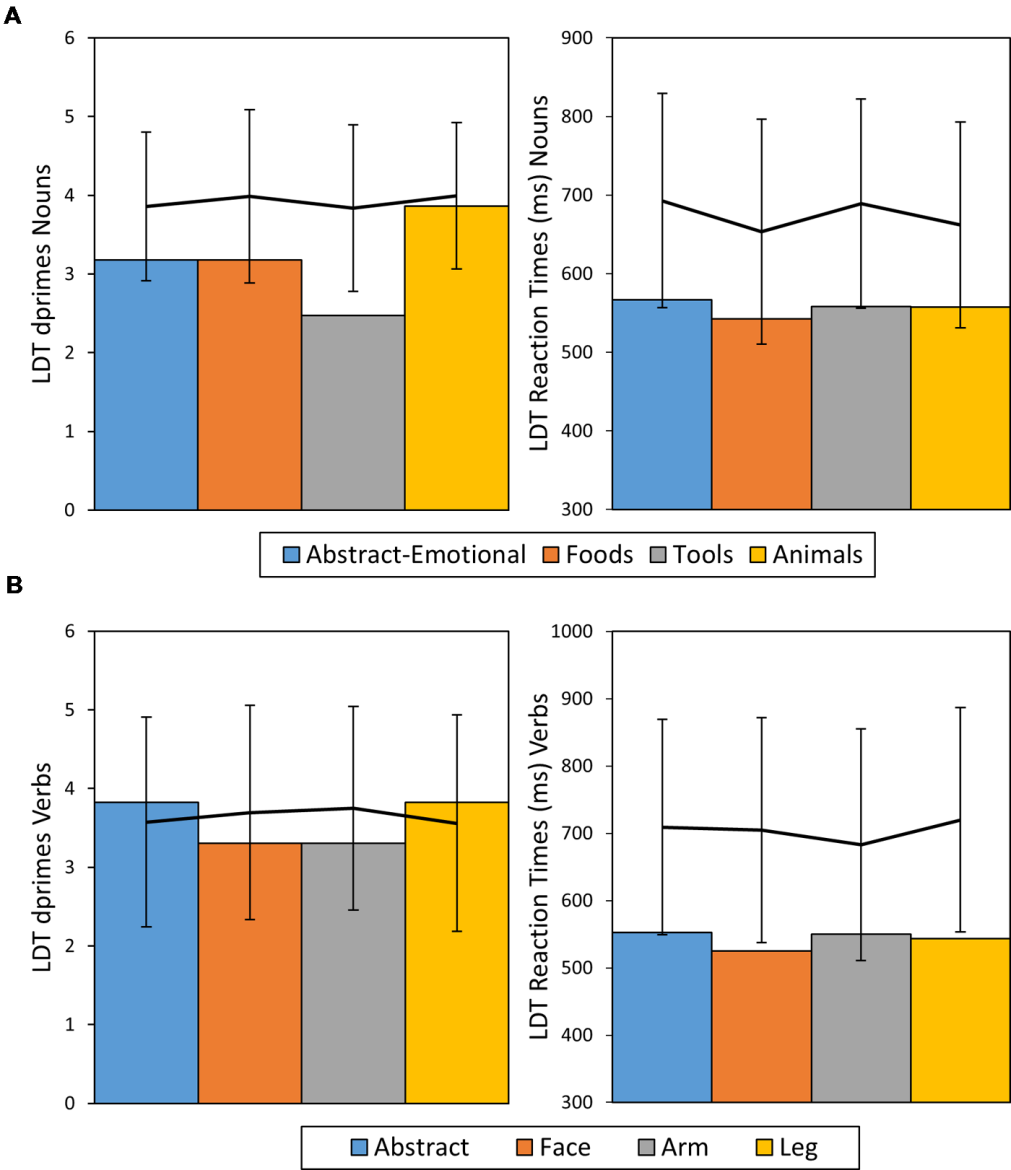
**Dprime and RT results of patient HS for nouns **(A)** and verbs **(B)** given in bar charts.** The line diagrams represent average performance of the control sample with error bars representing ± 2 SDs.

In direct comparison of noun and verb performance, HS exhibited no processing advantage for either word class in terms of ACC [ACC Nouns = 0.93, ACC Verbs = 0.95, χ^2^ = 0.5, *df* = 1, *p* = 0.48, Cramer’s *V* = 0.04] or RT [RT Nouns: *M* = 556 ms, *SE* = 6 ms; RT Verbs: *M* = 543 ms, *SE* = 8 ms, *t*(267.8) = 1.19, *p* = 0.23, Cohen’s *d* = 0.15]. A summary of HS’ LDT performance in comparison to results of healthy control participants can be found in **Figure 3.**

#### Patient CA

CA exhibited a strong impairment for the domain of abstract-emotional nouns, with an ACC of 0.43 while the other noun categories showed to be relatively intact in comparison (ACC = 0.78 on average). These differences were revealed to be statistically significant, both generally, between noun categories (χ^2^ = 19.15, *df* = 3, *p* <0.001, Cramer’s *V* = 0.37) and for the comparison of the abstract-emotional nouns versus the other categories combined (χ^2^ = 18.13, *df* = 1, *p* <0.001, Cramer’s *V* = 0.34), whereas *post hoc* comparisons for the other semantic noun categories yielded no significant differences (all *p* > 0.2, n.s.). Furthermore, performance on abstract nouns was also more error-prone than that on the non-action reference category of animal nouns (χ^2^ = 13.65, *df* = 1, *p* <0.001, Cramer’s *V* = 0.41). *Post hoc* RSDT results confirmed this observation, as the difference in ACC between animal and abstract nouns was significantly more severe in patient CA than in the control sample [*t*(20) = -2.05, *p* = 0.027, Z-DCC = -2.19] and likewise the comparison of abstract nouns vs. all other noun categories combined [*t*(20) = 3.15, *p* = 0.002, Z-DCC = -3.39]. Finally, even the pairwise χ^2^ noun category comparisons showed abstract word ACCs to be lower compared with each of the other noun categories (all *p* < 0.05, Bonferroni corrected), whereas the other noun groups did not significantly differ between each other.

**FIGURE 4:**
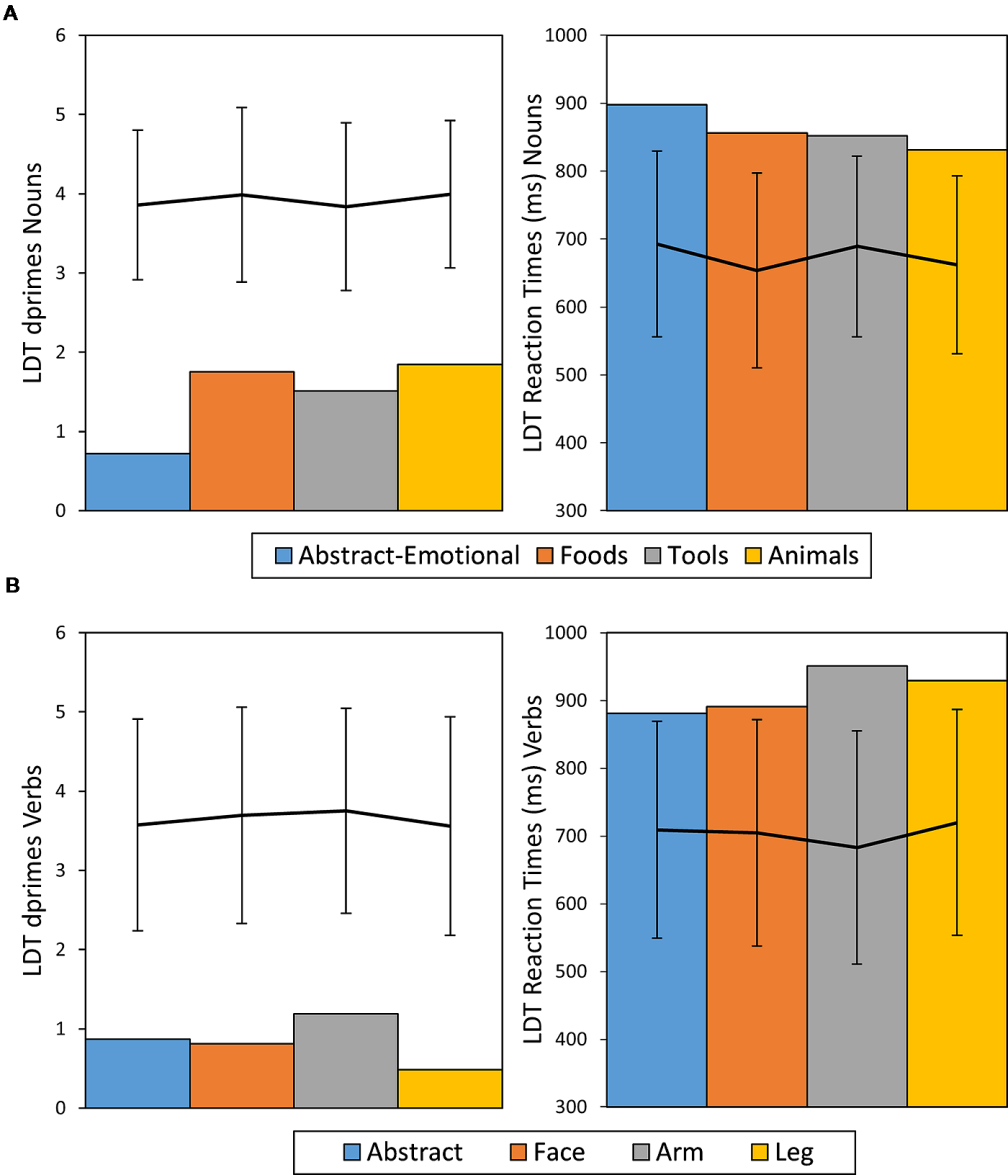
**Dprime and RT results of patient CA for nouns **(A)** and verbs **(B)** given in bar charts.** The line diagrams represent average performance of the control sample with error bars representing ± 2 SDs.

For verbs, overall ACC was poor across categories (0.46 on average) and differences between categories were not significant (χ^2^ = 6.13, *df* = 3, *p* = 0.11, Cramer’s *V* = 0.2). Analysis of RTs did not show significant effects of semantic word category in either nouns[*F*(3,103) = 0.78, *p* > 0.2, η*^2^* = 0.02, n.s.] or verbs [*F*(3,67) = 0.71, *p* = 0.2, η^2^ = 0.03, n.s.]. Across semantic categories, performance was worse for verbs, than for nouns, as measured by ACC (ACC Nouns = 0.69, ACC Verbs = 0.46, χ^2^ = 17.5, *df* = 1, *p* < 0.001, Cramer’s *V* = 0.23) and RT (RTs Nouns *M* = 853 ms, *SE* = 14 ms, Verbs *M* = 913 ms, *SE* = 20 ms, *t*(176) = 2.5, *p* = 0.01, Cohen’s *d* = 0.38). Taking the healthy participant sample as a benchmark, RTs and ACCs were considerably impaired across all noun and verb categories in patient CA, with all measures being outside of the range of ± 2 SDs from the mean of the control sample. **Figure 4** provides an overview of CA’s LDT results in comparison to performance of healthy control participants.

#### *Post Hoc* Matching of Semantic Categories for RTs

Despite the careful matching of word stimuli for psycholinguistic features, RTs in healthy control subjects happened to differ significantly between semantic categories within nouns and verbs classes. To investigate whether this RT difference may affect the patterns of category specificity seen on ACC data in our patients, an additional *post hoc* stimulus matching was performed, now using average RTs in the healthy control cohort as an additional matching criterion. This was done by removing 20% (i.e., eight) of the items of each semantic noun category, those with the shortest average RT for foods and tools and the 20% slowest items for abstract and animal nouns. The resulting item set did no longer show significant RT differences in the healthy controls [By-subjects: *F*(3,60) = 1.19, *p* > 0.2, η*^2^* = 0.06; by-items: *F*(3,124) = 1.02, *p* > 0.2, η*^2^* = 0.02] while, the previously reported category-specific patterns in the patients’ ACC data could be confirmed for the same item selection on χ^2^ and RSDT measures, with patient CA showing the selective deficit for abstract emotional nouns compared to the other categories [χ^2^ = 5.4, *df* = 1, *p* = 0.02, Cramer’s *V* = 0.21; RSDT: *t*(20) = 1.95, *p* = 0.03, *z*-DCC = -2.1] and patient HS exhibiting a selective impairment for tools compared to the other semantic noun categories [χ^2^ = 6.4, *df* = 1, *p* = 0.02, Cramer’s *V* = 0.22; RSDT: *t*(20) = 2.75, *p* = 0.01, *z*-DCC = -2.96].

## Discussion

Two patients with focal lesions in their dorsal frontocentral primary, premotor, and supplementary motor areas participated in standard aphasia tests as well as a speeded lexical decision paradigm. Albeit general aphasia measures, including comprehension tests, did not indicate neurological language disorders, LDT results of both patients revealed differential impairments of semantic categories of nouns. In patient CA, who suffered from a focal lesion of the left SMA, this impairment was most pronounced for abstract-emotional nouns, whereas patient HS, who suffered from a mild paresis of the right extremities and focal lesion just inferior to the typical hand representation in the primary motor cortex, showed a category-specific deficit in recognizing tool-related nouns. Both observations show that the motor system can be necessary for recognizing and processing of words from specific semantic categories. HS’ data confirm a necessary role of motor cortex for action-related tool word processing and CA’s results show that motor systems, especially the SMA, can be of relevance for abstract-emotional symbols. These results refute the hypothesis that motor brain areas play merely an epiphenomenal role in processing words with action-related and abstract meaning. It is clear that our present results emerging from the performance patterns of two patients cannot motivate general conclusions on all patients with similar lesions. Single case studies as the ones presented provide the existence proof that category-specific action-semantic deficits can arise from motor system lesions and this observation can be computed against the predictions of established semantic brain theories, as discussed below.

### Category-specificity, General Cognitive Deficits, and Lesion Localization

Our proposed conclusions on category-specific semantic deficits imply that the observed performance pattern cannot be a result of general cognitive or linguistic impairments in the patients. Clinical language performance revealed by AAT results showed almost errorless performance and therefore demonstrates absence of aphasia. In particular, the excellent results obtained by both patients on the subtest on Word Comprehension show good general reading skills, which are important for written word and pseudo-word processing required in the LDT. Despite the absence of aphasia, both patients exhibited impairments in the LDT, arguably due to its higher processing load, especially the strict time constraints and emphasis on accuracy, compared with clinical testing with the AAT battery, where speed is not an issue. One might still argue that, possibly, additional general cognitive deficits, e.g., in praxis, attention, memory, or planning might have been present in the patients, but remained undetected and may have affected the results. However, general cognitive deficits can be expected to lead to reduced performance across semantic word categories. In contrast, the processing deficits observed in both patients, which were significantly most pronounced for one semantic word category, argues against an explanation in terms of general cognitive defects and in favor of one emphasizing specific and semantic origins. It is possible that the overall very poor LDT performance across all semantic categories seen in CA was due to the functional role of the affected SMA and adjacent pre-SMA in decision and motor response selection (Hernández et al., 2002; Forstmann et al., 2008; Nachev et al., 2008); however, again, the fact that this deficit was most pronounced for abstract-emotional nouns cannot be explained by such a general cognitive processing impairment.

Given the etiology of the lesions in CA and HS, it may seem that the lesions might not entail the focality needed to draw conclusions about the functional roles of specific brain areas, as proposed in the introduction. As discussed by Karnath and Steinbach (2011), it might be problematic to precisely tell apart functional and non-functional tissue in brain tumor patients. Furthermore, Karnath and Steinbach (2011) also highlight the possibility that gradual functional reorganization may continuously occur during the extended period of tumor growth, thus compensating for the impaired functionality. Both objections would resemble a blurring of the inferences that can be drawn on the functional role of lesioned brain areas. With regard to the former objection, it has to be noted that HS showed a rather circumscribed lesion and patient CA’s metastasis (in contrast to other tumors like for example gliomas) did allow for fine grained differentiation between lesioned and non-lesioned tissue. In addition, the disadvantage of a possibly poor spatial resolution of causal inferences on the functional role of brain areas is not unique to tumor patients, but indeed resembles a general problem for all kinds of lesion studies (Shallice and Skrap, 2011). This has been investigated in detail, for example in the context of reperfusion of the ‘penumbra’ of stroke-related lesions (Hillis et al., 2006). Similarly, the argument of better functional restitution in tumor patients does not apply to the current cases, as a category-specific deficits were in fact manifest and detectable using psycholinguistic methods in both patients, whereas functional reorganization would have predicted absence of such specificity. Even if functional reorganization occurred in any of the patients, it can be assumed to be insufficient to recover normal function so that a functional role of the lesioned brain areas can still be soundly derived (Duffau, 2011). However, we should remark that for many other patients, the argument is still valid and significant category-deficits may not arise from motor systems lesions. Functional reorganization provides one important reason why category differences may be frequently absent after focal lesions.

With regard to the lesion in patient HS, it has to be noted that although ADEMs are most often diagnosed with multiple lesion foci (Karussis, 2014; Koudriavtseva et al., 2015), cases with monofocal lesions have been reported on multiple occasions (Kesselring et al., 1990; Miller et al., 1993; Murthy et al., 1999), allowing to assume a focal etiology. In patient CA, who suffered from a circumscribed metastasis, areas adjacent to the lesioned SMA, including pre-SMA and primary and pre-motor cortex, may have been affected in their function. We should, however, draw attention to the fact that the patient’s tumor had been subject to intensive therapy previous to testing, including partial extirpation, so that it appears unlikely that pressure was exerted on adjacent areas. Still, the possibility that partial lesion of pre-SMA played some role in causing the deficit in abstract noun processing cannot be ruled out with certainty based on our present data.

Clinical observations are consistent with the claim that patient CA, but not HS, was suffering from depressive symptoms at the time of testing, although this could not be objectified using psychological tests. Intuitively, this depressive mood could be seen as a (non-neurological) reason for the processing deficit for abstract-emotional words. However, previous studies either indicated that LDT performance was not affected by depression (Clark et al., 1983; Challis and Krane, 1988) or even led to a facilitation of LDT performance on emotionally congruent word stimuli (Olafson and Ferraro, 2001). Note that most of the abstract emotion words used in the present study were negative in valence and therefore congruent with the negative emotional state of depression. The observed performance reduction for abstract emotion words seen in patient CA contrasts with these earlier observations, rendering it unlikely that the observed category-specific semantic word processing deficit was based on emotional state of the patient at the time of language testing.

Apart from the neurological and clinical factors mentioned above, one could try to argue that the specific impairments found in the two patients might in fact not be due to compromised processing of word semantics, but rather to impairments of basic visual or linguistic processing. It is well-known that, in order to solve the LDT, it is not necessary to engage semantic processing, because words, but not pseudo-words, are familiar entities stored as whole lexical entries in the brain-internal ‘mental lexicon’. Nevertheless, the LDT paradigm has previously been shown to be sensitive to manipulation of semantic content (James, 1975; Chumbley and Balota, 1984; Kroll and Merves, 1986; Jin, 1990; Samson and Pillon, 2004) and a range of pre-existing neuropsychological studies demonstrated category-specificity in processing semantic word categories after focal brain lesions (Gainotti, 2010). In the present study, the examined semantic word categories, within each greater lexical category, were meticulously matched for a range of psycholinguistic features, including word length, lemma frequency, character, bi- and trigram frequencies and their word-initial counterparts, as well as number and word frequency of orthographic neighbors. Therefore, the observed category-effects can soundly be attributed to differences in word semantics and not to sub-lexical, morphological or other psycholinguistic properties, some of which have previously been shown to modulate the activity of motor areas during language processing, independent of semantics (Pulvermüller et al., 2006; de Zubicaray et al., 2013). In addition, the close matching of words and pseudo-words with regard to character, bi- and trigram frequencies as well as word initial character and bigram frequencies, argues against the possibility that sublexical strategies played a role in the present LDT, instead of actual semantic processing of target stimuli.

### Category-effects Across Participants, Measures, and Lexical Classes

In contrast to the category-specific patterns shown by both patients with focal lesions in the motor system, *d*′ and ACC data showed that the healthy control population performed similarly on all semantic noun categories and the same applied for the matched verb categories too. However, semantic category differences may be suggested by the control subjects’ response time data, which yielded significant differences due to slightly slower responses to abstract and hand-action related nouns. These were the two categories, respectively, affected in our patients. To examine the theoretical possibility that the processing difference suggested by controls’ RT data may explain the category specific patterns in our patients, analyses were repeated with a subset of the word stimuli matched for response times in healthy controls. The RT-matched semantic word category sets did not yield any significant performance difference in our healthy subjects, neither in ACCs nor in RTs, but the category-differences for semantic noun categories in both patients’ ACC values were reconfirmed. These results rule out the possibility that, whatever might have caused the RT differences in our control population could explain the category differences seen in the patients.

In both patients, the category specific impairments were only found for nouns, but not for verbs. This observation might appear surprising, as the majority of previous studies on motor semantics highlighted the role of the sensory-motor systems for the processing of action verbs. Considering the stimuli selected for the present LDT though, one cannot conclude from this result that the functional role of motor areas applies exclusively to the processing of nouns. The experimental setup was designed to compare processing of semantic categories separately within semantic subtypes of nouns and, again, for subtypes of verbs. Because psycholinguistic matching was not performed across noun and verb categories, a direct comparison between the lexical classes is not straightforward. For example, verbs had higher lemma frequencies than nouns and therefore were more familiar. This implies that the LDT was generally easier for verbs compared with nouns. At the same time, pseudo-verbs consistently differed in only one syllable from proper verbs (because of the shared suffix ‘-en’), whereas nouns differed between each other in both of their syllables, thus making it necessary to process more information for making lexical decisions on nouns than on verbs. In addition, it is well known that verbs carry more syntactic information and are generally more strongly action-related semantically but are, on the other hand, less imageable than nouns (Pulvermüller et al., 1999; Bird et al., 2000). Some of these differences between the lexical categories (e.g., the greater imageability of nouns) may underlie the observed processing advantage of nouns over verbs, as found in the healthy controls’ d’ and RT results and in CA’s reduced performance on all verb categories. These general psycholinguistic differences between nouns and verbs may also in part account for the fact that category differences could only be documented for one of the lexical categories, because a difference on one of the psycholinguistic dimensions may have moved one of the categories away from a ceiling or floor so that performance differences could become selectively manifest.

While patient HS’ overall performance for verbs on the LDT was comparable to that of healthy controls, results for CA revealed a strong impairment across all verb categories, which was only paralleled by the severely affected abstract word category of nouns. Being aware of the mentioned psycholinguistic differences between our lexical class stimuli, we should still mention the possibility that the latter observation could, in theory, originate from the relatively higher relevance of action knowledge for the semantics of verbs. From an Embodied Cognition perspective, the observed impairment for all verb categories with action dominant semantics seem to fit to CA’s lesion site in the left SMA, an area known to be involved in motor planning independent of motor effector and body part (Roland et al., 1980; Fried et al., 1991). Nevertheless, given that potential differences in task difficulty cannot be ruled out when comparing nouns and verbs, this interpretation has to be treated with caution before less ambiguous experimental evidence is available. In the case of patient HS, the fact that no semantic category effects were seen for verbs could be seen as a side effect of the high performance close to ceiling for verbs, whereas average performance on nouns was relatively reduced. Our data did not show significant differences in processing different semantic sub-categories of verbs, thus confirming the corresponding observation by Arevalo et al. (2012). To disentangle the possible factors influencing verb and noun performance, future studies should aim to match semantic categories between those grammatical word classes in terms of semantic features, psycholinguistic characteristics as well as general task difficulty. However, we once again remind the reader that such matching is not trivial and might be not possible on all dimensions (for discussion, see Bird et al., 2000; Neininger and Pulvermüller, 2003).

### Relationship of the Present Results to Known Neuropsychological Dissociations

The reported selective impairment for tool nouns in patient HS adds to previous findings on impairments in neurological patients, specifically for words with action related semantics (Bak et al., 2001, 2006; Neininger and Pulvermüller, 2001, 2003; Pulvermüller et al., 2010; Arevalo et al., 2012; Kemmerer et al., 2012). In contrast to these earlier works, the current study shows that those selective impairments can be induced by rather small focal lesions (of 18 mm diameter in the case of HS) in the motor areas and confirms that the corresponding category-specific semantic deficits are not restricted to action-related verbs but can also arise for nouns used to speak about objects that afford actions, as for example tool words. HS’ results on tool nouns also fit well with the results of earlier neuro-stimulation experiments, which pointed out the functional relevance of motor areas for action verb processing, using facilitatory (Pulvermüller et al., 2005a) or virtual lesion approaches (Willems et al., 2011), although in those studies effects were found solely on RTs. As substantial numbers of errors were here documented to arise from motor system lesion for nouns with action-affording referents, the present results show a necessary role of motor and premotor cortex in one single neurological case. Over and above previous research, we show a rather narrow level of category-specificity, in so far as it applied only to nouns used to speak about objects affording actions typically performed with the hand. This specificity is consistent with semantic somatotopy in the motor system (Pulvermüller, 2005; Pulvermüller and Fadiga, 2010).

Observations on the performance of patient CA on the other hand revealed a functional involvement of supplementary motor systems also for the processing of abstract-emotional nouns, which lack the transparent sensory-motor components of their concrete counterparts. This can be seen as first evidence that activity in motor areas during the processing of abstract-emotional nouns, as revealed by earlier fMRI results (Moseley et al., 2012), does in fact not resemble an epiphenomenon, but an integral part of word comprehension instead, which is necessary for optimal word processing. This result appears consistent with semantic grounding theories postulating involvement of motor circuits in abstract semantic processing, thus suggesting that the ‘embodiment’ does not necessarily need to limit its scope to the processing of words referring to concrete entities. At the theoretical level, there is indeed motivation to see an intrinsic connection between abstract-emotional meaning and the bodily actions with which such meanings are expressed (for discussion, see Barsalou and Wiemer-Hastings, 2005; Moseley et al., 2012; Pulvermüller, 2013). Whether this holds exclusively for abstract-emotional words, or renders an effect that is valid also for non-emotional abstract symbols and concepts, has to be determined by future studies, for example by investigating stimuli across different subcategories of abstract words.

### Distributed Semantic Circuit Account of the Current Results

In order to explain the category-preferential semantic deficit in processing hand-action-affording and abstract-emotional nouns, one may claim that our results are consistent with theories that view the motor system as the main carrier of meaning processing for these specific semantic types. Although such strong statements – that motor cortices but no other areas integrate concepts and word meanings – have hardly been made, some arguments against semantic grounding (e.g., in Mahon and Caramazza, 2008) seem to focus on this hypothetical position. Indeed, some authors have stated “that the modalities of action and perception are integrated at the level of the sensorimotor system itself and not via higher association areas” (Gallese and Lakoff, 2005, p. 459), and such statements may have laid the ground for the idea that motor systems, but not association or convergence zones such as the prefrontal or anterior-temporal cortex, might carry meaning. Although even such a strong postulate about semantic integration in motor but no other multimodal brain systems could indeed be strengthened by the present data, it is not the only position that explains the present results. Considering a wider spectrum of data, which also show semantic activation of and semantic deficits after lesion in multimodal areas (e.g., Patterson et al., 2007; Binder et al., 2009; Vigliocco et al., 2014), the more appropriate explanation of the present data needs to be phrased in terms of distributed semantic circuits in which neurons in motor areas play a functional, causal and necessary role.

In this perspective, the sensorimotor parts of the distributed semantic circuits would carry aspects of word meaning and contribute to a process of immediate ‘simulation’ of semantic information (in the sense of Jeannerod, 2006) when symbols are perceived, even if subjects do not actively attend to them (Pulvermüller et al., 2005b; Shtyrov et al., 2014). Therefore, the observations made in both patients on nouns seem to fit especially well into theoretical frameworks that assume distributed cell assemblies with different cortical distributions to be the basis of semantic processing of words (Pulvermüller, 1999). Those cell assemblies are assumed to be the result of correlational learning mechanisms driven by Hebbian learning principles (Hebb, 1949). If a word often co-occurs with specific sensory and or motor experiences, or likewise with specific sensory or motor imagery, that word’s semantic circuit would gradually be represented by a distributed cell assembly reaching into the sensory or motor areas where relevant activations had been present. A word like “hammer” co-occurring with performance, perception or imagery of specific motor movements afforded by the tool, would co-activate the perception action circuit for the word form and the action-related neuronal circuit, thus yielding a higher-order distributed semantic circuit in which neurons in motor areas take a causal and necessary functional role. This proposal does not postulate a unique role of the motor system (or ‘modality specific cortices’) as a seat of semantics, but a semantic role of cortical circuits distributed over perisylvian, sensorimotor and multimodal convergence areas. Specificity in cortical function arises from the fact that, for different meaning types, these semantic circuits have different cortical distributions – with some (action related) semantic circuits, but not others (non-action related ones), reaching into the motor system. Importantly, in this view, the word ‘hammer’ is not exhaustively semantically processed in multimodal areas, as postulated by disembodiment (or weak ‘integrative’) approaches to semantics, and there is no preferential status of the motor system for semantics either. Semantic circuits for abstract-emotional words would include neurons in the limbic system – because emotional-affective ‘inner states’ are essential for at least some abstract words (Meteyard et al., 2012) – and in the motor system – because the learning of at least some abstract-emotional words requires the grounding of word forms in emotions expressed in overt body movements (Moseley et al., 2012). This integrative action perception model appears to us to be consistent with known lesion results on brain-lesion-elicited semantic impairments (Kiefer and Pulvermüller, 2012; Pulvermüller, 2013) and to do best justice to the present data.

## Conclusion

Category-specific semantic deficits in a LDT seen in two patients with focal lesions in their left hemispheres reveal the functional necessity of primary/pre- and supplementary motor areas for the processing of concrete hand-action affording as well as for abstract-emotional nouns. Processing of concrete tool nouns was selectively impaired after lesions of hand motor cortex, while a lesion in the left SMA resulted in impaired processing of abstract-emotional nouns.

## Conflict of Interest Statement

The authors declare that the research was conducted in the absence of any commercial or financial relationships that could be construed as a potential conflict of interest.
